# Obstructive fibrinous tracheal pseudomembrane: Sudden child death following laser removal of papillomata

**DOI:** 10.1002/ccr3.5346

**Published:** 2022-02-10

**Authors:** Caroline Rhame, Janette Verster, Johan Dempers, Pierre Goussard

**Affiliations:** ^1^ Division of Forensic Medicine Department of Pathology Faculty of Medicine and Health Sciences Stellenbosch University and Tygerberg Hospital Cape Town South Africa; ^2^ Department of Paediatrics and Child Health Faculty of Medicine and Health Sciences Stellenbosch University and Tygerberg Hospital Cape Town South Africa

**Keywords:** inflammatory pseudomembrane, laryngeal papillomatosis, obstructive fibrinous tracheal pseudomembrane, sudden death, upper airway obstruction

## Abstract

Obstructive fibrinous tracheal pseudomembrane (OFTP) is a rare complication usually following endotracheal intubation, occurring when a collection of inflammatory exudate coalesces at the site of damaged epithelium within the trachea and along the tracheal mucosa, creating a luminal narrowing and subsequent airway obstruction.

## Graphical abstract

Endotracheal intubation and endotracheal procedures can cause pseudomembrane formation. This is a rare complication and should be considered in patients presenting with respiratory failure or atypical respiratory symptoms post extubation. This includes laser surgery for the removal of viral papillomata in children.

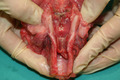

## 1 INTRODUCTION

Obstructive fibrinous tracheal pseudomembrane (OFTP) is a rare complication usually following endotracheal intubation, occurring when a collection of inflammatory exudate coalesces at the site of damaged epithelium within the trachea and along the tracheal mucosa, creating a luminal narrowing and subsequent airway obstruction.^1^ OFTP should be considered in the presentation of post‐extubation respiratory symptoms, particularly in the case of post‐extubation stridor or respiratory failure. Although uncommon, this condition is potentially life‐threatening and may require immediate recognition and definitive emergency airway management to prevent asphyxia.

We report a case of acute upper airway obstruction and sudden death in a child with inflammatory pseudomembrane formation after laser surgery for laryngeal papillomatosis.

## 2 CASE REPORT

A 6‐year‐old female with sudden onset of stridor was seen at a tertiary hospital in Cape Town, South Africa, and a diagnosis of laryngeal papillomatosis was made. Emergent laser surgical removal of the papillomata was performed for management of the upper airway obstruction. The patient was intubated with difficulty at the time of surgery with a size 4.0 mm cuffed endotracheal tube (ET) due to the virus papillomata obstructing the airway. The cuff was not inflated. After an unremarkable surgical course and subsequent admission to the post‐operative pediatric intensive care unit (PICU), the patient was extubated on day one post procedure. The duration of intubation was 24 h. She had persistent stridor post extubation, which improved on adrenalin nebulization.

She was discharged from PICU and was reported to be playing that evening. Fifteen hours after discharge from the PICU, the patient experienced a sudden and unexplained bout of coughing and subsequent collapse. The patient could not be successfully intubated and further resuscitation attempts were unsuccessful.

**FIGURE 1:**
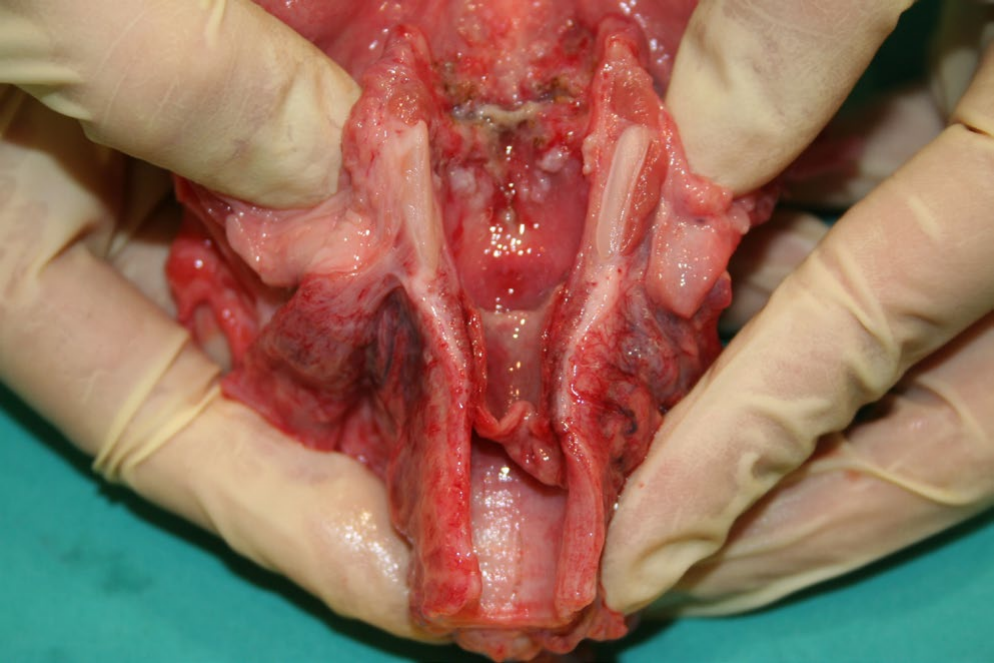
This figure depicts the larynx and trachea at autopsy, with the surgical bed visible in the glottis and subglottic region, and the extensive inflammatory pseudomembrane visible in the proximal trachea, ±30 mm below the true vocal cords. This pseudomembrane measured ±25 mm in length

**FIGURE 2:**
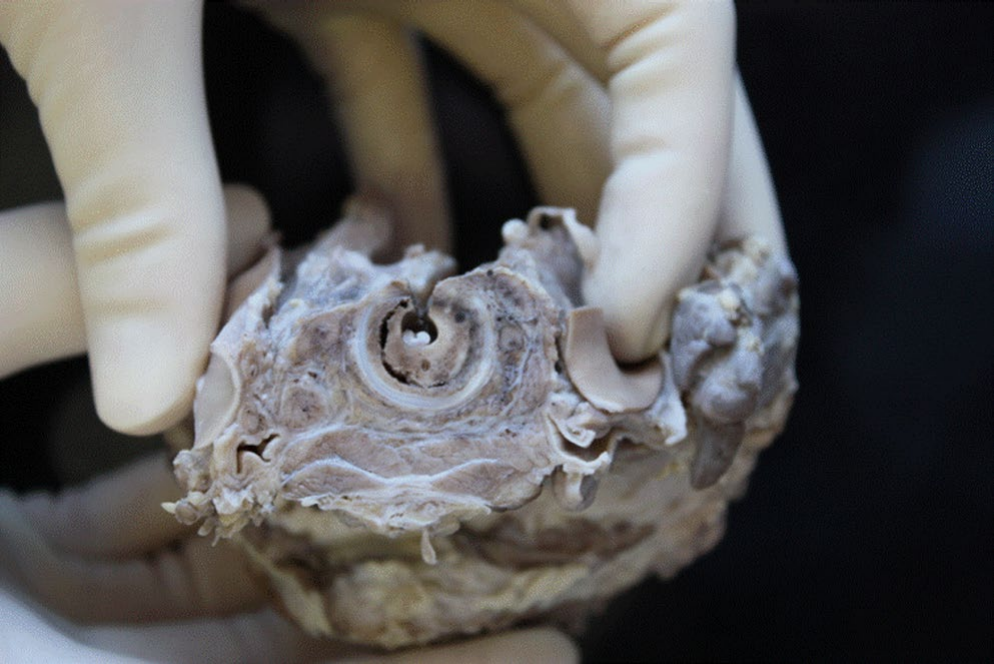
This figure shows a cross section of the proximal trachea, including the inflammatory pseudomembrane, after it had been fixed in formalin. The inflammatory pseudomembrane extends circumferentially around the mucosa of the trachea, almost completely occluding the lumen of the trachea

**FIGURE 3:**
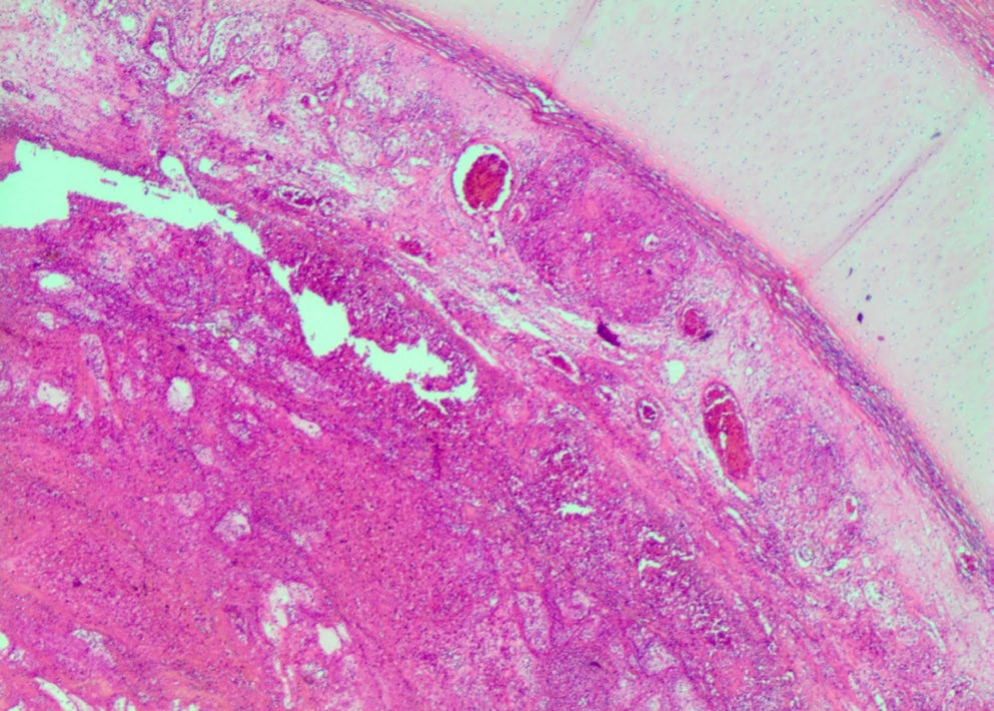
This figure exhibits a histological section of the trachea and inflammatory pseudomembrane. Tracheitis was clearly present, with a partially adherent inflammatory exudate. Abundant granulation tissue was forming, and neovascularization was further observed in the affected region

**FIGURE 4:**
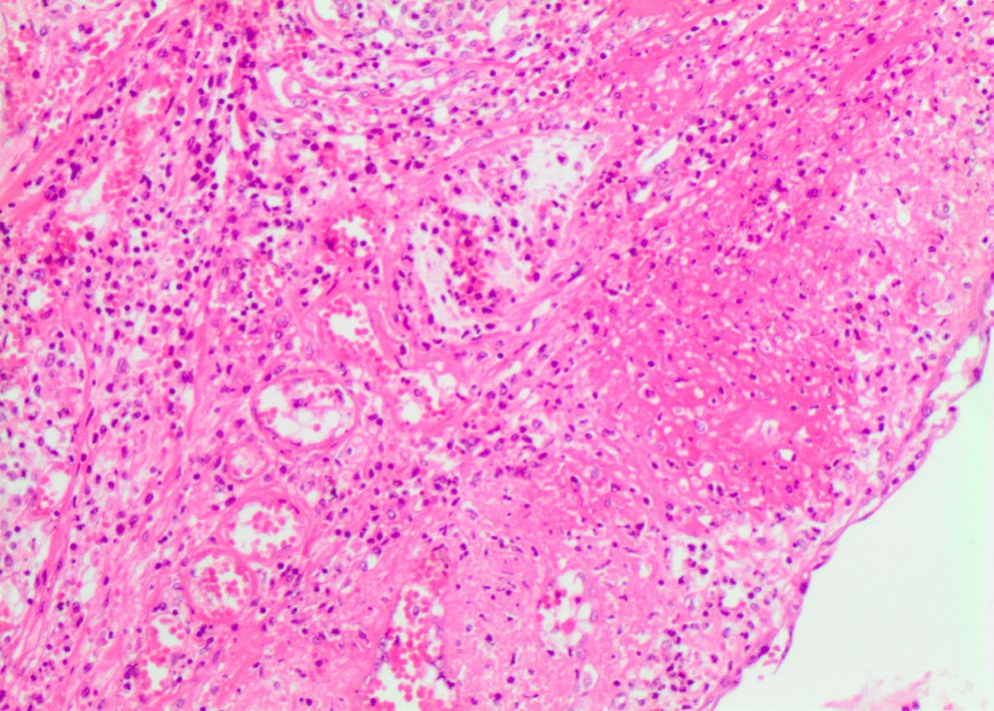
These histological sections depict the inflammatory pseudomembrane, which greatly decreased the aperture of the lumen of the trachea. The pseudomembrane consisted of a dense, acute inflammatory exudate, including abundant neutrophils, red blood cells, entrapped mucous, as well as fibrin layering

**FIGURE 5:**
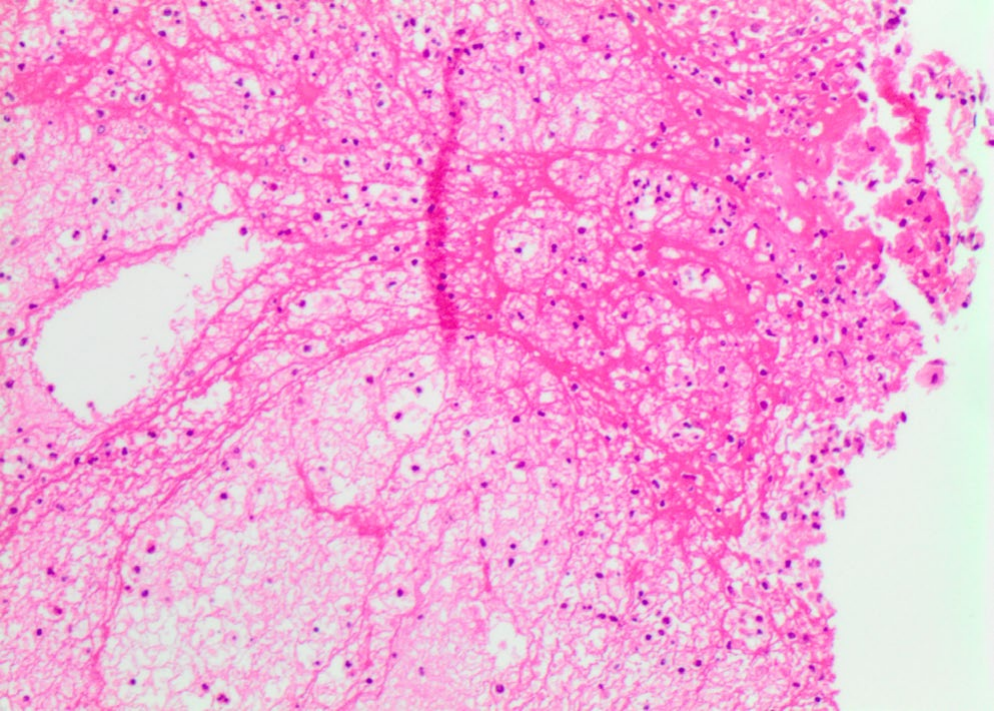
H&E histological staining clearly showing fibrin layering within the inflammatory pseudomembrane

At autopsy, a macroscopic, fibrinous, purulent tracheal membrane of approximately 25 mm length lined the tracheal mucosa circumferentially, approximately 30 mm inferior to the true vocal cords, with hyperemic mucosa surrounding the airway (Figure 1). The distal end of the pseudomembrane was loosened from the tracheal mucosa and folded proximally, resulting in complete occlusion of the tracheal lumen (Figure 2). Histological examination revealed an intense tracheitis with a dense inflammatory exudate and fibrin deposition (Figure 3, 4, 5).

Additional findings included the remnants of polypoid masses along the glottal mucosa, both superior and inferior to the true vocal cords, with overlying mucosal changes consistent with surgical laser burns.

These findings were consistent with sudden and catastrophic upper airway obstruction and sudden death due to asphyxia, following formation and partial detachment of a fibrinous inflammatory pseudomembrane, most likely as a consequence of a coughing fit.

## 3 DISCUSSION

OFTP in itself is a rare complication of endotracheal intubation, with 54 cases of both adult and pediatric patients reported in a systematic review by Sehgal et al, where varying case characteristics were reported, including diverse indications for intubation, age of the patient, type of the endotracheal tube used, and the time intubated.^2^ The median duration of intubation for the pediatric patients was 35 h.

The risk factors are not well documented, and various hypotheses exist to explain possible etiopathogenesis. Previous literature suggested that formation of the pseudomembrane may be linked to ischemic necrosis of tracheal mucosa although more recent publications have not correlated pseudomembrane formation to high pressure cuffs, and various case reports exist of pseudomembrane formation in pediatric patients where the cuff was not inflated.^2^, ^3^

A second hypothesis of tracheal injury, particularly by gastric acid secretions and subsequent insult to the epithelium due to vomiting, traumatic intubation, or aspiration is also suggested.^3^, ^4^ Other suggested contributing factors include necrosis due to systemic hypotension, such as in the case of sepsis or cardiopulmonary resuscitation, or coexisting microvascular pathology, as is seen in diabetes.^1^, ^4^

It is not known how commonly pseudomembrane formation co‐occurs in the context of direct surgical manipulation of the tracheal mucosa, including carbon dioxide laser removal of papillomata, and whether this confers increased risk of pseudomembrane formation. It is also not known if keeping the patient intubated or if the duration of intubation after surgical intervention prevents this complication. In the literature, this phenomenon is not specifically described as a complication of carbon dioxide laser surgical removal of papillomata, both after an initial surgery and after repeating surgical interventions, though glottic webs and other soft tissue complications are well documented and specifically related to surgical technique and laser emission parameters.^5^

The clinical presentation of OFTP is diverse and correlated to the degree of airway obstruction, with respiratory symptoms such as stridor, cough, hoarseness, and respiratory failure appearing from immediately post extubation to as delayed as 70 days after endotracheal tube removal.^1^, ^2^ Sehgal et al described a median interquartile range of 6–96 h, though noted that the onset of symptoms was more rapid in their pediatric cohort.^2^ Atypical presentations such as positional respiratory failure due to a ball valve effect of a pseudomembrane flap has also been described.^4^ The complication of sudden onset, unexpected death in infants with pseudomembrane is rare.

In several case reports, the emergency management of respiratory symptoms with steroids or adrenalin therapy, both inhaled and nebulized, is described as initially effective, though this effect appears transient in nature and worsening of the clinical condition may follow rapidly, as demonstrated in our illustrative case.^2^, ^3^, ^4^

As late or missed diagnosis is associated with increased mortality, and the complication of sudden detachment with acute airway obstruction is a possibility, rapid reintubation or rigid bronchoscopy may be lifesaving in the case of respiratory failure. Both flexible and rigid bronchoscopy are reportedly used successfully to confirm the diagnosis and to remove the pseudomembrane, often resulting in a rapid and complete resolution of symptoms.^1^, ^2^, ^3^, ^4^ Recurrence is rare.^2^

Written informed consent was obtained from the patient's parent to publish this report in accordance with the journal's patient consent policy.

## 4 CONCLUSION

Both endotracheal intubation and endotracheal procedures have the potential to cause pseudomembrane formation. We present the first case of OFTP in a young child without prolonged intubation post laser surgery for the removal of viral papillomata.

Although this is a rare complication, all those involved in care of intubated patients and endotracheal interventions should be suspicious of this diagnosis in any patient who presents with respiratory failure or atypical respiratory symptoms post extubation. Rapid diagnosis and airway management may be lifesaving.

## CONFLICT OF INTEREST

None.

## AUTHOR CONTRIBUTIONS

CR and PG: were responsible for creating the manuscript and editing. JV and JD: were responsible for managing the case and creating the manuscript.

## ETHICAL APPROVAL

The case report was approved by the Human Research Ethics Committee of Stellenbosch University (HEA‐2020‐18528).

## CONSENT

Written informed consent was obtained from the patient's parent to publish this report in accordance with the journal's patient consent policy.

## Data Availability

Not relevant.
